# Flexible Femoral Nailing of an Awake Adult Muscular Dystrophy Patient

**DOI:** 10.5435/JAAOSGlobal-D-19-00013

**Published:** 2019-12-24

**Authors:** Matthew J. Brown, Kim Song, Mary Vollmar, Marcus Shelby, Holly Leshikar

**Affiliations:** From the Davis Department of Orthopaedic Surgery (Dr. Brown, Dr. Shelby, Dr. Leshikar), University of California, Sacramento, CA; University of Miami (Ms. Vollmar), Coral Gables, FL; and the Davis Department of Anesthesiology and Pain Medicine (Dr. Song), University of California, Sacramento, CA.

## Abstract

Femoral fractures can be common in nonambulatory patients with myopathies because they present with notable osteoporosis. From the orthopaedic perspective, this can be complicated by a pre-existing knee flexion contracture and small femoral shaft size. The goals of treatment are to reduce external immobilization, maximize comfort for transfers, prevent functional loss, and preclude refracture. The purpose of our work is to describe the anesthetic and orthopaedic considerations in treating a bed-bound adult patient with nemaline dystrophy and a midshaft femur fracture. The authors have obtained the patient's informed written consent for print and electronic publication of the case report.

There is a known and acknowledged risk in dystrophic patients of fractures.^1,2^ Most recent efforts have focused on the preventive treatment of fractures in the cohort through glucocorticosteroids,^3^ calcium and vitamin D supplementation,^4^ and even the use of bisphosphonates.^5^ However, there has been little published about appropriate surgical care of long-bone fractures in the nonambulatory dystrophic cohort, aside from an article by Huber et al, discussing the flexible intramedullary nailing of femur fractures in 6 patients with myopathies,^6^ and an article by Biber et al^7^ describing 3 cases of retrograde nailing. Here, we describe the flexible nailing of a femur fracture in an awake adult dystrophic patient.

## Case Report

**Figure 1:**
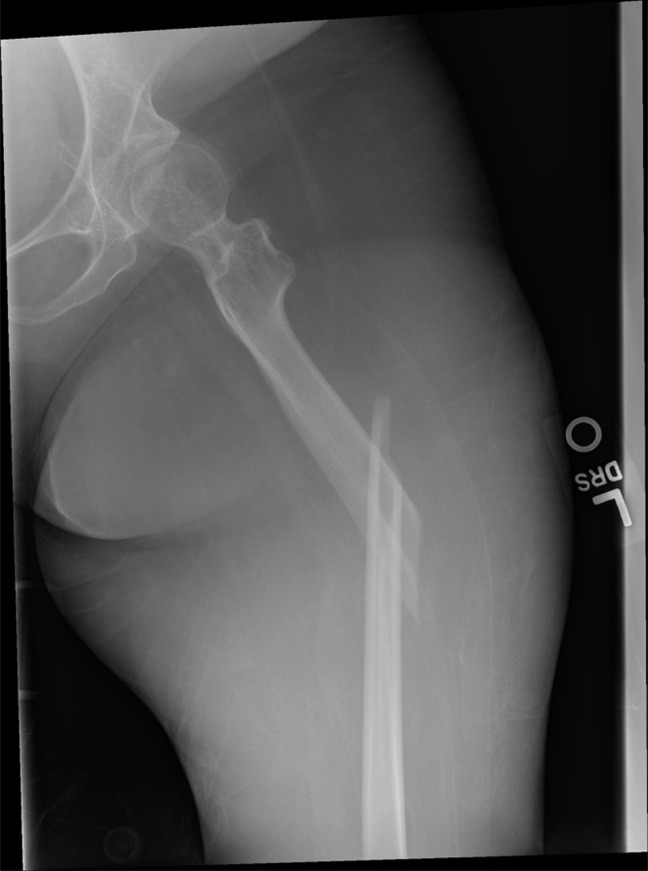
Preoperative radiographs demonstrating midshaft femur fracture in patient with miniscule diaphyseal size.

The patient is a 26-year-old woman with a body mass index of 17.23 and a history of nemaline muscular dystrophy who was bed-bound at baseline. She presented as a transfer to our emergency department with a report of an audible crack and left leg pain after transferring off of a bed pan on the day of presentation. In the emergency department, the patient was diagnosed with a midshaft left femur fracture (Figure 1), and an orthopaedic consultation was obtained from the adult orthopaedic trauma service. Given her nonambulatory status, osteopenia, and a miniscule femoral shaft diameter, the initial recommendations were for a trial of nonsurgical treatment.

By hospital day 2, the patient was noted to be uncomfortable without adequate pain control subject during positioning, hygiene, and toileting. The pediatric orthopaedic service was consulted and subsequently determined that the patient's status, especially for movement and transfers, could be markedly improved through fracture fixation. However, the diameter of the patient's femur was a concern, as it was discovered that no commercially available femoral nail would fit both the curve and diameter of her femoral shaft. Instead, fracture fixation through flexible nailing was decided to be the optimal technique.

Restrictive lung disease secondary to the patient's nemaline rod myopathy and scoliosis required her to be on 24-hour BiPAP at baseline. Because she had previously remained on prolonged postoperative ventilatory support for three days after a general anesthetic, it was her expressed wish that she not undergo airway manipulation for this procedure to avoid the possibility of long-term ventilator support. A spinal anesthetic was considered, but ruled out in favor of a regional anesthetic due to the patient's severe scoliosis, impalpable landmarks, and procedural positioning difficulties. Instead, an ultrasound-guided femoral nerve block was done with the patient in supine position.

Monitored anesthesia care with sedation was provided in addition to local lidocaine injected in the metaphyseal distal femoral start points to supplement areas not covered by femoral nerve block. The patient was maintained on her usual BiPAP regimen throughout the procedure. Because the patient was accustomed to a high-dose opioid regimen at home (liquid methadone and acetaminophen-codeine through gastric tube), she required similarly high doses intraoperatively. Despite these doses, the patient remained conscious and received emotional support throughout the procedure.

After appropriate sterile preparation and draping, it was determined fluoroscopically that the fracture had shortened roughly 3 cm. The fracture was gently pulled out to length, and traction was maintained. The lateral incision was made under fluoroscopic guidance and carried to and then through the iliotibial band.

**Figure 2:**
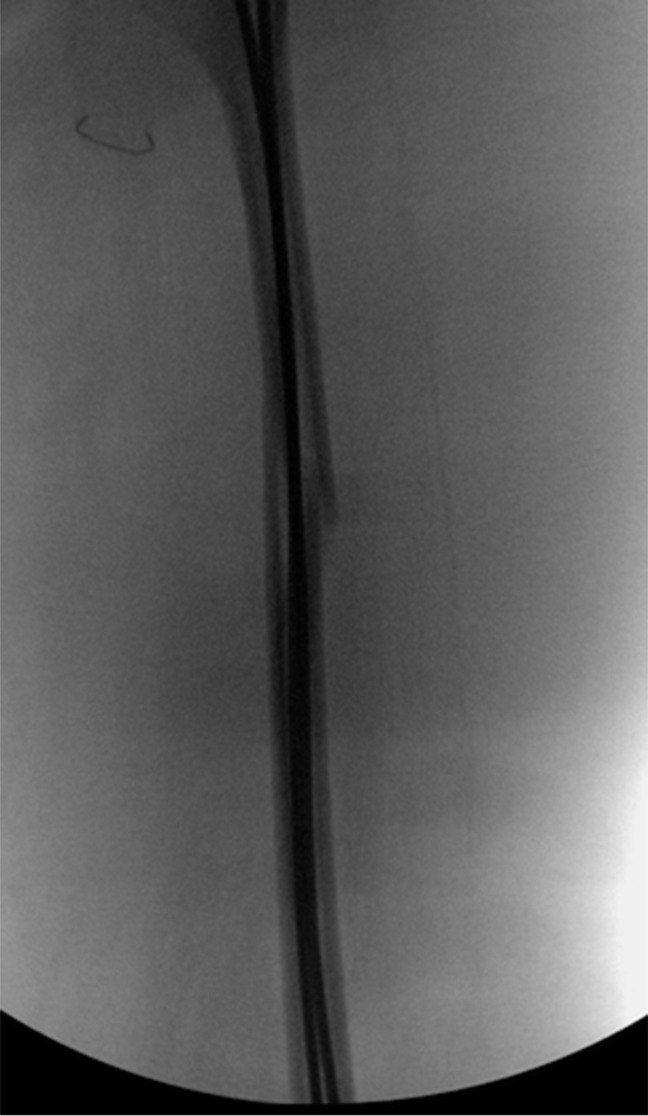
Intraoperative fluoroscopy images demonstrating flexible nail fixation of midshaft femur fracture.

Under fluoroscopic guidance, an awl was used to form an oblique hole in the metaphysis. A prebent 2.5-mm flexible nail was then inserted and passed to just below the fracture site. Then, a medial incision was made at the level of the metaphysis, the vastus medialis was retracted anteriorly, and the awl was again used to broach the cortex in an appropriate metaphyseal location. The precontoured 2.5-mm flexible nail was then placed and brought to the level of the fracture. Then, using a combination of traction and the F-tool, we were able to reduce the fracture and advance both flexible nails to the level of the subtrochanteric region. After fluoroscopic evaluation, we were satisfied with our reduction. The exposed parts of the nails were cut at the skin level and then advanced another centimeter, so that their final position was below skin level and not prominent (Figure 2). The iliotibial band, subdermal tissue, and skin were then closed.

**Figure 3:**
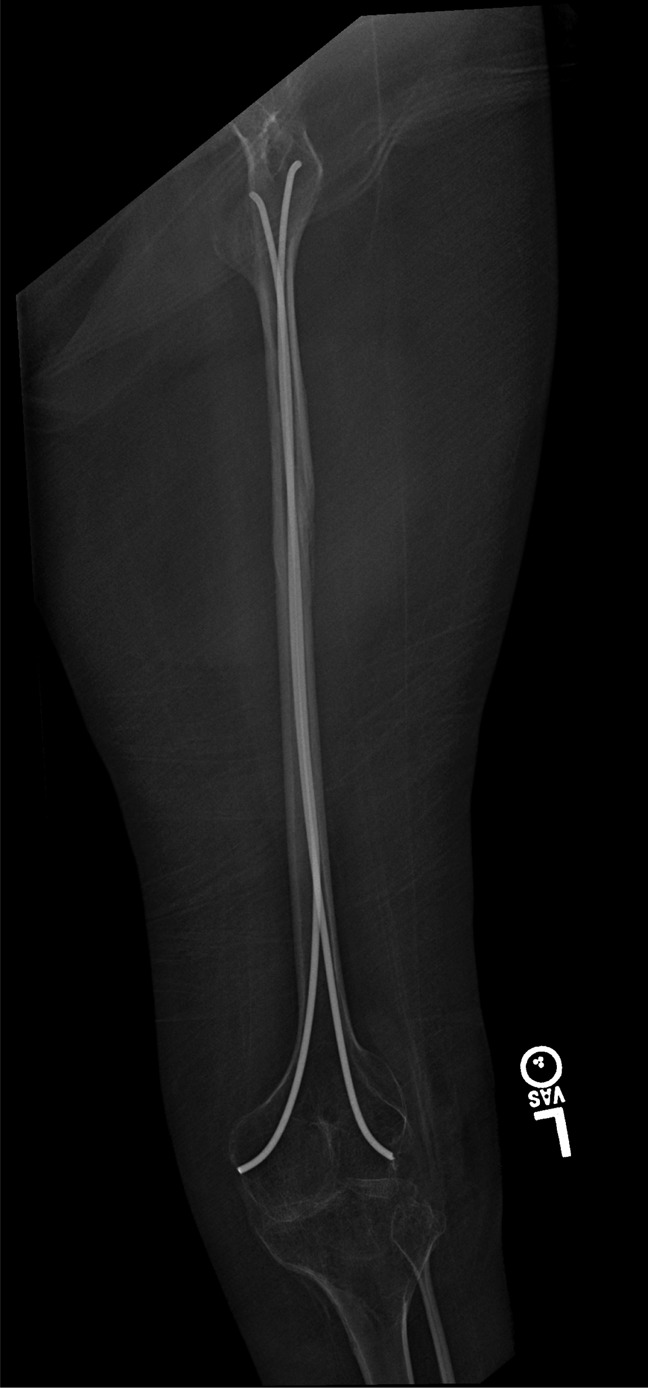
Six-month postoperative radiographs demonstrating full fracture healing with retained implant.

Postoperatively, the patient remained in the hospital for 3 days. She was non–weight-bearing on that limb, but was much more comfortable with transfers even immediately postoperatively. After 2 weeks, she was seen in clinic, where her wounds were healing well and her radiographic images demonstrated maintained position of implant and interval healing. She continued to be seen postoperatively until full fracture healing was demonstrated (Figure 3). Her main reports were occasional lateral hip and knee pain noted most when she was rolled onto that side. However, the dramatic pain noted with transfers before the operation resolved.

## Discussion

It is understandable that for many years, primary treatment of fractures in nonmobile dystrophic patients has relied on primarily nonsurgical modalities.^1^ A number of studies documented good outcomes in nonambulatory dystrophic patients with long bone fractures treated nonoperatively.^1,2^ However, the presence of a painful, poorly controlled femoral fracture has previously been used as justification for surgical fixation of both adult^8^ and pediatric dystrophic patients.^9^

Owing to complicated medical implications, even the most common dystrophies may require multiple services including but not limited to cardiology, pulmonology, endocrinology, neurology, and physiatry to be able to care for the patient before, during, and after surgery. The variety of presentations of the greater than 800 separate entities that fall under the classification of neuromuscular disease is extensive. In this type of setting, it is important to enlist an anesthesiologist prepared to address not only the specific type of dystrophy, but additionally, any accessory conditions with which the patient may present.

When considering anesthetic management for patients with nemaline rod myopathy, there are notable risks to every major organ system, depending on the anesthetic technique selected. From a pulmonary standpoint, respiratory muscle and diaphragm weakness pose the greatest risk, limiting administration of benzodiazepines, opioids, and neuromuscular blocking agents.^10^ This, in turn, complicates pain control and ventilatory management during general anesthesia. Depending on the type of sedation used, regional anesthesia can preclude these risks to the respiratory system.^8^ There have also been cardiologic and endocrine abnormalities described in the literature that deserve attention preoperatively with dystrophic patients, in addition to physical and occupational therapeutic concerns that should be addressed.^11^

The reasoning behind using flexible intramedullary nailing in this case was primarily focused on the small diameter of the patient's femoral diaphyseal shaft. Owing to not ambulating for over 15 years, the patient's femoral shaft was too small to accept a commercially available static locking nail. Instead, we had to rely on intramedullary flexible nailing, a technique used most successfully in smaller (<100 lb) children.^11^ However, recently, more data have emerged on the applicability of flexible nailing in the heavier (>100 lb) patient, and it has been as successful as the lighter cohort.^13^ This methodology has previously been verified for quicker mobilization in children,^14^ so with the limitations imposed by our patient's condition, we felt that flexible nailing could stabilize her fracture while allowing for less pain with transfers. The mindset on the feasibility of flexible nailing in the pediatric cohort has been focused on the adaptability of the nail diameters to the narrow canal size coupled with the improved ability of children to heal fractures based on secondary bone healing. The ability of flexible nails to provide fairly rigid fixation at the femoral fracture isthmus while remaining minimally invasive appears to allow for adequate healing despite the turning necessary for daily care in a patient such as ours.^6^

Alternative surgical methods have been noted to have lower rates of success in young patients with osteopenia due to inherited or genetic conditions. Often, plate and screw constructs do not provide sufficient stability and the bone is prone to refracture above or below the level of a plate.^15^ Rigid locked intramedullary nails have a high risk of taking a false route in osteoporotic bone, can cutout through weak bone, and can result in the interlock screws tearing out of the bone.^6^ In this scenario, we felt that flexible nailing would provide the best fixation.

The use of flexible nailing also allowed for minimal anesthetic usage, which went along with both our patient's wishes and the data on successful fracture treatment in this cohort.^8^ It also is revolutionary in that, in the previous literature, there exist little data on lower extremity reduction and fixation with minimal anaesthesia.^8^ The data on pediatric fracture reductions under minimal anesthesia focus on upper extremity fractures and demonstrate worse results with patients older than 10 years.^16^ As has been previously demonstrated, the older patients are, the more they catastrophize fracture reduction and they react accordingly.^17^

In conclusion, our report demonstrates that the use of awake flexible nailing of femoral shaft fractures even in nonambulatory dystrophic patients is feasible, reliable, and a minimally invasive method. We believe that surgical treatment with flexible intramedullary nailing should be discussed in those patients who need regional or local anesthesia only for fracture reduction and should certainly be considered in those patients where minimally painful mobilization is difficult or impossible with splints or casts.
